# Sex-related differences in Huntington‘s disease: a scoping review

**DOI:** 10.1186/s13293-026-00895-9

**Published:** 2026-04-03

**Authors:** Greta Hemicker, Katarína Schwarzová, Clancy Cerejo, Samuel Labrecque, Marina Peball, Federico Carbone, Bernadette Wimmer, Atbin Djamshidian, Klaus Seppi, Beatrice Heim

**Affiliations:** Department of Neurology, Medical University of Innsbruck, Innsbruck, Austria

**Keywords:** Huntington’s disease, Sex-related differences, Rare neurodegenerative disease, Genetics, Clinical phenotype, Biomarker

## Abstract

**Background:**

Huntington’s disease (HD) is an autosomal-dominant neurodegenerative disorder and emerging evidence indicates sex-specific differences in disease manifestation and progression.

**Objective:**

To summarize current evidence on sex-related differences in HD across genetic, clinical, biomarker, and treatment domains and identify gaps in knowledge.

**Methods:**

A literature search was performed in PubMed and Google Scholar (up to 31.01.2026). Eligible studies examined sex- or gender-related differences in individuals with genetically confirmed HD or those at risk. Meta-analyses, reviews, animal studies, theses, abstracts only, and non-English papers were excluded. After screening and full-text review, secondary citation tracking identified further studies. *Sex* was defined as the biological classification of participants (male/female) as originally reported, whereas *gender* was defined as socially constructed roles or identities, where explicitly assessed. Terminology in this review reflects the terminology used in the original publications.

**Results:**

Fourty-four studies met inclusion criteria. Genetic studies showed similar cytosine-adenine-guanine (CAG) repeat lengths across sexes when parent-of-origin was not considered, while paternal transmission was consistently linked to repeat expansion and earlier disease onset. Clinically, women more frequently exhibited depression, irritability, and greater functional impairment, whereas men more often showed apathy and substance misuse. Medication patterns differed, with women being more likely to receive antidepressants and anxiolytics, and men antipsychotics. Biomarker studies indicated potential sex-related differences in body composition, uric acid, neuroimaging, and hormonal profiles, though available evidence remained heterogeneous and exploratory.

**Conclusions:**

Sex-related differences in HD are evident in genetic transmission, psychiatric symptoms, functional decline, and medication use. Biomarker findings suggest additional sex-specific biological signatures.

**Supplementary Information:**

The online version contains supplementary material available at 10.1186/s13293-026-00895-9.

## Introduction

Huntington’s disease (HD) is an autosomal-dominant inherited neurodegenerative disorder. Cardinal symptoms include movement disorders with typical chorea, behavioral abnormalities such as depression or apathy, and cognitive decline, particularly executive dysfunction, impairment in processing speed and emotion recognition, which increases with disease progression [1]. The disease is caused by a repeat-expansion of the cytosine-adenine-guanine (CAG)-triplet coding for the Huntingtin-Protein, that is engaged in neuronal transport mechanisms and cell-to-cell interaction. The mutated Huntingtin-Protein (mHTT) is unable to perform its regular designated tasks which leads to further accumulation and ultimately neurodegeneration [2]. Age of onset and symptom severity are inversely correlated with CAG-repeat length, however CAG repeat length does not entirely explain their variability [3]. Distinct sex-specific differences in disease prevalence, such as the higher incidence of multiple sclerosis in women or Parkinson’s disease in men, are not commonly observed in autosomal-dominant inherited disorders like HD [4, 5]. Some studies, however have shown that there are indeed sex-related differences other than prevalence, for example regarding clinical presentation [6, 7, 8].

Sex- and gender-informed research is increasingly recognized as essential for understanding and managing HD. Emerging clinical data suggest that biological sex may influence disease manifestation, course and treatment needs in clinically meaningful ways [9, 10]. In this review, we summarize and critically appraise current evidence on sex-related differences in HD and highlight their implications for clinical practice and research.

## Methods

### Population

This systematically conducted scoping review included studies evaluating sex or gender differences in individuals with genetically confirmed HD at various stages of the disease, or studies published prior the development of direct mutation testing according to established clinical criteria [11, 12, 13, 14]. The review focused on sex- or gender-stratified analyzes in the domains of epidemiology, genetics, clinical presentation, medication use, and biomarkers. Studies were required to report results separately for male and female participants or to explicitly analyze sex or gender as an independent variable.

In this review, sex was defined as the biological classification of participants as female, or male as reported in the original studies. Gender was defined as socially constructed roles or identities, where explicitly assessed. However none of the included studies stated a clear definition of the words sex and gender, therefore we assumed that both words were used as synonyms for the biological sex. The terminology in this review reflects the terminology used in the original publications.

### Search strategy and search yield management

The review was conducted in accordance with PRISMA-ScR guidelines. An electronic search was performed in PubMed and Google Scholar on 16 February 2026 at 09:30 and 18:00 CET respectively using the Chrome web browser. PubMed Searches employed the advanced search interface and were restricted to English-language publications. Google Scholar results were sorted by relevance and limited to English-language publications as well.

In PubMed, the search term (“Huntington Disease“[MeSH]) AND (sex[tiab] OR gender[tiab]) yielded 519 records. Google Scholar returned approximately 9960 results using the search terms “Huntington disease” AND (“sex differences” OR “gender differences”). Given the limited reproducibility and feasibility of screening the full Google Scholar yield, the first 500 records ranked by relevance were screened. Across both databases, 1019 records were identified in the search. After removal of 25 duplicates, 994 records underwent title and abstract screening, and 948 were excluded. 46 articles were assessed for full-text eligibility. In addition, 18 studies were uniquely identified through forward and backward citation tracking. Altogether 64 full-text articles were assessed for eligibility, of which 20 were excluded. In total, 44 studies met all eligibility criteria and were included in the present scoping review.

All records from both databases were imported into Rayyan (www.rayyan.ai) for deduplication and blinded screening (*n* = 519 + 500=1019). Duplicates were identified using Rayyan’s automated detection tool and manually verified where necessary (*n* = 25). Two reviewers (GH, CC) independently and blinded to each other’s decisions screened titles and abstracts (*n* = 994). Afterwards, a decision was made on which articles should be included in the full-text review. In cases of uncertainty, the record was included for full-text assessment to ensure completeness(*n* = 46 + 18=64). Disagreements were resolved by discussion, with consultation of a third reviewer (BH) if required. Data extraction was performed by one reviewer (GH).

### Eligibility criteria and quality assessment

Studies were included if they (1) enrolled participants with genetically confirmed HD or at risk for HD (asymptomatic HD mutation carriers), or if published prior the development of direct mutation testing according to established clinical criteria (2) reported original research data, (3) examined outcomes in relation to sex or gender, and (4) presented sex- or gender-stratified analyses or explicitly tested sex or gender as a variable. Studies were excluded if they (1) were animal trials, theses, books, abstracts only, meta-analyses or reviews, (2) were case reports with fewer than three participants, (3) reported sex distribution but did not analyze outcomes by sex, or (4) did not provide accessible full text. We did not include systematic reviews or meta-analyses to only analyse primary evidence in this scoping review. To assess the quality of each included study we applied the Joanna Briggs Institute Critical Appraisal Tools (JBI). Two investigators (GH, CC) independently evaluated all studies and any disagreement was resolved consulting a third investigator (BH). The highest available score was 11 points for cohort studies, 8 points for cross sectional studies, 10 points for case series and case-control studies. Overall quality of studies was examined as follows: high quality (≥ 70% of overall score), medium quality (50–70% of overall score) and low quality (≤ 50% of overall score).

### Data charting and extraction

A standardized data-charting form was used. Extracted variables included first author, year of publication, sample size, sex distribution, disease stage, variables assessed, and sex-specific results.

## Results

After excluding duplicates, a total of 64 studies, published between 1 January 1988 and 31 January 2026 were screened in full length. Studies were then analyzed according to first author, year, region, study design, sample size, sex division, variables measured and results per sex. Finally, the review included 44 studies examining sex- or gender-related differences in HD (see Table 1). These studies addressed various topics, including epidemiology, genetics, clinical presentation, medication use, and biomarkers.

**Table 1:** Included studies

Topic	Author	Year	Sample	Sex division (F/M)	Variables measured
Epidemiology	Bruzelius et al.	2019	3707	2094/1611	Epidemiology of HD in the USA
Genetics	Aziz et al.	2011	337 parent-offspring pairs	191 maternal, 146 paternal	Factors associated with CAG repeat instability during intergenerational transmission
	Cannella et al.	2004	57 juvenile HD		Effect of sex of transmitting parent
	Dale et al.	2016	453	250/203	Disease stage and sex as predictors of anxiety and depression in HD
	Farrer et al.	1992	1764 HD individuals		Effects of parental age, birth order, parental age at onset, and sex of the affected (grand)parent
	Foroud et al.	1999	2068	1074/994	Relationship between age at onset and illness duration
	Goldberg et al.	1993	21		Sequential events underlying HD
	Hentosh et al.	2021	2145	1097/1048	Sex-related differences in clinical HD
	Kehoe et al.	1999	133	77/56	APOE genotype as a modifier of age-at-onset variation in HD
	Kremer et al.	1995	254 affected parent-child pairs with HD 440 parent-child pairs with CAG size in the normal range	135 maternal, 119 paternal	Intergenerational CAG repeat changes in the HD gene
	Myers et al.	1991	42	23/19	Factors influencing slow progression, effects of treatments and substance use, and expected HD progression rates
	Nørremølle et al.	1995	65 parent-offspring pairs		Correlation between repeat instability and parental repeat length
	Ranen et al.	1995	277 parent-offspring pairs		Intergenerational behavior of the trinucleotide repeat and its relationship to anticipation
	Roos et al.	1991	2617	1263/1354	Effect of parent and patient sex on age of onset
	Saft et al.	2004	145 HD		Relationship between age at onset of HD and Apolipoprotein E genotypes
	Taylor	2005	57 individuals at risk of HD	38/19	Gender differences in attitudes toward genetic risk and predictive testing in individuals at risk for HD
	Telenius et al.	1993	42 juvenile HD	23/19	Relationship between trinucleotide expansion and juvenile onset in HD
	Trottier et al.	1994	36 unrelated HD families		Instability of CAG repeats in relation to parental transmission and age at onset
	Weydt et al.	2014	1706		associations between PPARGC1A genotype and age at onset in HD
	Wheeler et al.	2007	112 sperm DNAs from male HD gene-positive 184 father-offspring* 311 mother-offspring pairs**	*96/88 **147/164	Factors associated with repeat instability
	Zielonka et al.	2008	41 HD mutation carriers	23/18	Correlation between CAG repeat length, age at onset, symptom profile, disease progression, and gender
	Zielonka et al.	2013	1267	636/631	Role of gender in phenotypic expression and progression of HD
	Zielonka et al.	2018	2191	1080/1111	Gender differences in motor, cognitive and behavioral symptoms, functional impact, and quality of life
	Zühlke et al.	1993	352	193/159	CAG repeat length
	Chen et al.	2024	269	155/114	Temporal trends of symptoms in individuals with HD in China
Clinical presentation	Ekkel et al.	2023	173	87/86	Patient and disease characteristics, functioning, and gender differences in HD patients living in nursing homes
	Epping et al.	2013	803 HD mutation prodromal; 223 mutation negative	512/291 143/80	Depression in prodromal HD: prevalence, severity, clinical correlates, and relation to time since genetic testing
	Hemicker et al.	2026	102	58/44	Sex differences in clinical presentation, functional outcomes, employment status, and use of supportive therapies
	Levkova et al.	2025	43	24/19	Clinical and genetic aspects of HD in a single-center cohort
	Marder et al.	2000	960	499/461	Rate of functional decline
	Philpott et al.	2016	15 premanifest; 11 symptomatic; 16 HC	25/17	Influence of gender on cortical inhibitory deficits
	Van Duijn et al.	2010	152 HD mutation carriers 56 non-carriers	84/68 31/25	Prevalence and clinical correlates of apathy in HD
Medication Use	Feleus et al.	2024	8546	4461/4085	Medication use in HD patients
	Rowe et al.	2012	787 prodromal HD 215 HC	499/288 139/76	Patterns of antidepressant use in a prodromal HD
Biomarker: Fluid/Body composition	Corey-Bloom et al.	2020	69 Plasma 94 Saliva	40/29 55/39	Uric acid levels in peripheral fluids and postmortem brain tissue in HD and their clinical associations
	Costa De Miranda et al.	2019	21 early-stage HD 29 healthy controls	11/10 14/15	Body composition parameters, bone mineral density, and early biomarkers of weight loss in HD
	Goodman & Barker et al.	2011	25 premanifest HD 25 sex, age, ethnicity matched HC	15/10 15/10	Change in body composition in premanifest HD
Neuroimaging	Lee et al.	2017	75 at risk 136 healthy controls	39/36 69/67	Effects of subthreshold CAG repeat length on brain structure and function in children at risk for HD
	Looi et al.	2012	17	6/11	Differential putaminal morphology as a marker of clinical dysfunction
	Sampedro et al.	2021	41	21/20	Influence of NfL on brain atrophy and clinical severity
	Yao et al.	2025	19,738	10,763/8975	Interplay of sex and CAG-age product score on clinical measures and neuroimaging biomarkers
Hormonal	Markianos et al.	2005	42 HD patients 44 HC	42 M	Plasma testosterone, LH, and FSH levels and their associations with disease symptomatology in genetically confirmed male HD patients
	Markianos et al.	2007	41 HD patients 18 females with expanded CAG repeats (at risk) 66 HC	41 F	Plasma levels of total testosterone, DHEAS, and cortisol and their associations with disease symptomatology in genetically confirmed female HD patients
	Saleh et al.	2010	109 HD		Somatotropic axis abnormalities as predictors of cognitive dysfunction

HD Huntington’s Disease; CAG Cytosine-Adenine-Guanine; Nfl Neurofilament light chain; LH Luteinizing Hormone; FSH Follicle-Stimulating Hormone; DHEAS Dehydroepiandrosterone Several studies addressed more than one area, so their placement within this table should not be interpreted as a strict or exclusive categorization

### Quality assessment

The result of the quality assessment is displayed in Supplement Tables 3, 4, 5 and 6. The majority of included studies resulted in high quality 7% had medium quality and none of the studies had low quality.

### Epidemiology

The incidence and prevalence of HD differ between regions and countries around the globe, resulting in a pooled incidence of 0.48 per 100,000 person years and a pooled prevalence of 4.88 per 100,000 [15]. One study further examined the incidence and prevalence rates of the disease specifically for the USA, with a focus on sex-related differences [16]. This resulted in an age-adjusted diagnostic frequency that was about 7.05/100,000 among women and 6.10/100,000 among men. No other study so far found a sex-related difference in prevalence or incidence rates among HD patients.

### Genetics

HD is an autosomal dominant disorder caused by a CAG repeat expansion in exon 1 of the HTT gene on chromosome 4. CAG repeat length can increase further in the next generation, a phenomenon known as anticipation, which is most often observed with paternal transmission [1].

Most studies report no significant sex difference in CAG repeat length [6, 7, 8, 17]. One report noted slightly higher mean CAG repeats in women, but this finding has not been replicated [18]. Associations between CAG repeat length, time since symptom onset, and UHDRS subscale scores have been described as stronger in women, while correlations between CAG length and age at onset appear in both sexes [19]. Multiple cohorts have examined sex-specific parent-of-origin effects and related genetic influences [20–24, 25, 26]. Across these studies, paternal transmission is consistently associated with greater CAG repeat expansions and earlier onset in offspring. Earlier work also reported that juvenile-onset HD, which often presents with parkinsonian rather than choreatic features, is more frequently linked to paternal inheritance, whereas maternal inheritance is more often associated with later onset [12, 27]. Some studies further suggested that advanced paternal age contributes to new mutations, although other work did not find a clear relationship between parental age and CAG repeat instability in offspring [21, 28, 29]. Additional analyses indicate that maternal transmission may show sex-specific repeat changes, with expansions more common from mother to son and contractions from mother to daughter [29].

Other studies reported differences in age at onset and disease duration depending on the sex of the affected parent, with paternal inheritance generally linked to earlier onset, and some parent-of-origin effects weakening after excluding juvenile cases [14, 30]. There is also evidence that maternal inheritance may be associated with a slower disease course in male offspring compared with paternal inheritance in male offspring [13]. Beyond repeat length, several candidate genetic modifiers have been reported with sex-dependent effects. Variants in PPARGC1A have been associated with earlier motor onset in men but not women, and ApoE-related associations with earlier onset have been reported in men in some work but not confirmed in others [17, 31, 32]. Finally, sex differences have also been described in how individuals engage with predictive testing and disclose genetic risk. Women have been reported to disclose risk more often and show greater coping tendencies, whereas men have reported greater concern about disadvantage and lower disclosure [33].

### Clinical presentation

Age at onset and clinical presentation in HD are influenced by genetic factors, including CAG repeat length, but these factors alone do not explain the sex-related differences observed in affected individuals. Across studies, findings on sex effects remain mixed, with some reporting associations with age at onset, disease progression, and symptom severity, and others finding no clear relationships [6–8, 18, 34]. Across neuropsychiatric and behavioral domains, several reports suggest that depressive symptoms and more severe motor impairment are more common in women, while men may perform better on cognitive assessments [8, 34–36].

In our cohort of a previous study, irritability was also reported more frequently in women [37]. Other work links apathy more strongly to men, and prodromal cohorts have reported associations between female sex and depressive symptoms before diagnosis [35, 38]. Reports also differ on the distribution of specific symptoms, with some describing depression and hallucinations as more frequent in women and substance misuse more common in men [6, 8, 18]. Even where overall psychological distress appears to be driven mainly by disease stage rather than sex, higher antidepressant use has been observed among women [18].

Studies focusing on functional capacity are likewise heterogeneous. Some analyses indicate greater impairment in activities of daily living, lower total functional capacity, and more severe functional decline in women, whereas others report similar scores in functional and behavioral scales [6, 8, 34, 39]. Another line of work suggests that motor symptoms may contribute more strongly to functional impairment in women, while cognitive symptoms show tighter associations with functional abilities in men [7]. In our cohort of a previous study, women had lower rates of full-time employment, while use of neurorehabilitation services did not differ between sexes [37]. Finally, a national survey study reported sex-specific temporal symptom trends, with women mainly showing increasing physical manifestations over disease duration, including weight loss, stiffness or inflexibility, oral or throat difficulties, impaired speech, loss of balance, and changes in handwriting, whereas men showed more pronounced increases in reduced concentration, psychotic symptoms, and suicidal ideation [40].

### Medication use

Sex differences in symptom profiles are likely reflected in pharmacological management in HD. Analyses from large observational cohorts, including Enroll-HD, have reported clear sex differences in overall medication exposure, with higher total medication use among women than men [41]. Women also appear more likely to receive antidepressants, particularly serotonergic agents, across disease stages and in both prodromal and manifest HD [42]. By contrast, men are more often treated with tetrabenazine and antipsychotics, commonly in the context of managing irritability and aggression, while treatment rates for chorea are reported to be broadly similar across sexes [41].

Emerging evidence further suggests that anxiolytics and analgesics may be prescribed more frequently to women.

### Biomarker

#### Fluid biomarkers

Recent reviews summarize several biofluid biomarkers associated with HD, including mutant huntingtin (mHTT and markers of neuronal injury such as total tau and neurofilament light chain (NfL) [43]. Beyond these molecular measures, some studies have explored physiological parameters linked to disease progression [44, 45]. Male HD patients generally exhibit lower body weight, body mass index (BMI), and lean body mass compared to female patients, whereas women show lower bone mineral density than both male patients and healthy controls [44, 45]. One study reported higher plasma and salivary uric acid (UA) levels in males across both premanifest and manifest HD [46]. Interestingly, UA levels decreased from premanifest to manifest stages only in female patients, suggesting a potential sex-specific biomarker of disease progression.

#### Neuroimaging biomarkers

Structural and functional imaging studies suggest that neurodegeneration in HD may follow sex-dependent trajectories. Some work reports that higher CAG repeat length relates to more preserved cortical and subcortical structure and higher cognitive measures in women, whereas in men increasing repeat length is associated with smaller striatal volumes and comparatively larger cerebellar volumes, consistent with divergent neurodevelopmental or compensatory patterns [47]. Other analyses have not detected sex differences in putaminal morphology, indicating that such effects may vary with disease stage or methodological approach [48]. Further studies examining interactions between sex and cumulative disease burden metrics such as the CAG-Age-Product (CAP) score describe smaller putaminal volumes in women but slower CAP-related striatal atrophy compared with men, while men show more pronounced cortical thinning, consistent with faster structural progression [49, 50]. Whether sex influences cortical inhibitory deficits was analyzed by one study which did not show any sex specific effects within the HD patient cohort [51]. Another study investigated the impact of NfL levels on brain atrophy an clinical progression, which was found to be more pronounced in female HD patients [52]. Despite this apparent structural preservation, women have been reported to show steeper decline in cognition and daily functioning on UHDRS-related measures and higher depression with lower apathy [53].

#### Hormonal biomarkers

Sex hormones may modulate both neurodegenerative processes and behavioral symptoms in HD. Experimental animal work supports a neuroprotective role of estrogen in preclinical HD models, although comparable effects have not been established in human HD trials [54, 55]. Clinical studies further suggest sex-specific associations between androgens and disease manifestations. In men with HD, plasma testosterone has been reported to be lower than in age-matched controls and to correlate negatively with disease severity and dementia measures, without clear associations with depression or psychosis [56]. In women with HD, testosterone and DHEAS levels have been reported to be similar to controls overall, but lower levels of both hormones were observed among women with depressive symptoms [57]. This pattern is consistent with a link between androgen status and mood symptoms in women, rather than cognitive impairment [57]. Beyond sex steroids, altered growth-related hormones have also been reported. Elevated growth hormone and insulin-like growth factor 1 levels have been associated with cognitive decline in men with HD, raising questions about a uniformly neuroprotective role of IGF-1 in this context [58, 59].

## Discussion

This scoping review highlights sex-related differences in HD across genetic, clinical, biomarker, and therapeutic domains (see Table 2). During the review process, heterogeneity across included studies became apparent. While domains such as epidemiology and genetics are well-investigated through large registry studies and systematic meta-analyses other areas like sex-specific differences in symptomatology and biomarkers rely on fewer studies with smaller cohorts. Although HD is an autosomal-dominant disorder that confers equal genetic risk to men and women, the presented evidence indicates meaningful sex-dependent variation in disease manifestation, progression, and medication use patterns. A persistent lack of attention to sex-related differences may limit the quality of clinical care and constrain the validity and generalizability of clinical research. Consistent with broader work demonstrating that systematic consideration of sex and gender improves diagnostics and therapeutic decision-making, these findings underscore the importance of explicitly incorporating sex as a biological variable in HD research and care pathways [60, 61].

**Table 2:** Scoping summary of sex-related differences in HD across key domains

Category	Feature	Summary	References
**Epidemiology**	Incidence and prevalence	No consistent sex difference in incidence or prevalence across populations	[15, 16]
**Genetics**	CAG repeat length	CAG repeat length does not differ substantially between sexes	[6–8, 17, 18]
	Parent-of-origin effects	Paternal transmission associated with greater repeat expansion, earlier onset, and juvenile HD; maternal transmission is more often associated with repeat stability/contraction and later onset, with some sex-specific maternal transmission patterns (e.g., expansions more often to sons).	[1, 12–14, 20–30]
	Genetic modifiers	Possible sex-specific effects of genetic modifiers: PPARGC1A variants may be associated with earlier onset in males; ApoE-related effects are inconsistent and not consistently replicated.	[17, 31, 32]
	Genetic risk disclosure and coping	Females report greater disclosure and more adaptive coping tendencies, whereas males report lower disclosure and greater concern about disadvantage.	[33]
**Clinical presentation**	Age at onset	Age at onset primarily determined by CAG repeat length rather than sex	[6–8, 18, 34]
	Motor impairment	Motor symptoms may contribute more strongly to functional impairment/decline in females, while association appear weaker in males	[7, 8, 34]
	Cognitive function	Males may perform better on some cognitive assessments; females may show steeper cognitive decline in some analyses, but findings remain heterogeneous.	[7, 8, 34]
	Psychiatric symptoms	sex-specific neuropsychiatric phenotypes are evident, with depressive symptoms, antidepressant use, irritability, and hallucinations more frequent in females, and apathy, aggression-related symptoms, substance misuse, psychotic symptoms, and increased suicidal ideation over time more frequent in males	[6, 8, 18, 34–38, 41]
	Functional capacity	heterogeneous results with greater impairment in activities of daily living, greater functional impairment, and lower employment rates in females	[6–8, 34, 37, 39]
	Symptom progression patterns	Sex-specific disease progression trajectories, with greater progression of physical and motor-related symptoms in females and greater progression of cognitive and psychiatric symptoms in males	[40]
	Body weight and composition	Sex differences in metabolic and skeletal profiles, with greater metabolic and catabolic burden, lower body weight, BMI, lean body mass, and higher bone mineral density in males compared to females	[44, 45]
	Overall medication exposure	Sex differences in medication use, with similar chorea treatment rates across sexes, but higher overall medication use in females, including more frequent antidepressants, anxiolytics, and analgesics, while males are more often treated with tetrabenazine and antipsychotics	[41. 42]
**Fluid biomarkers**	Sex hormones	Lower testosterone levels linked to depressive symptoms in females and greater disease severity and dementia in males, while potential neuroprotective effects of estrogen in preclinical models remain unconfirmed in humans	[54–57]
	Growth hormone and IGF-1	may contribute to disease progression in males	[58, 59]
	Uric acid	Potential sex-specific biomarker, with higher uric acid levels in males across premanifest and manifest stages and decreasing levels only in females over disease progression	[46]
	NfL	No consistent sex differences in NfL levels, with one study showing greater impact on brain atrophy and clinical progression in females	[52]
**Neuroimaging biomarkers**	Structural neurodegeneration	Inconsistent sex-dependent neurodegeneration trajectories across studies and disease stages, with one study showing CAG repeat length associated with greater structural preservation and cognitive measures in females but smaller striatal volumes and altered compensatory patterns in males, and another study showing slower CAP-related striatal atrophy but smaller putaminal volumes in females and more pronounced cortical thinning in males	[47–49, 51]

HD Huntington’s Disease; CAG Cytosine-Adenine-Guanine; BMI body mass index; IGF-1 Insuline like growth factor 1; Nfl Neurofilament light chain;

A central finding of this review is an overrepresentation of psychiatric symptoms in females with HD, particularly depression and irritability. Large observational studies and clinical cohorts indicate that depressive episodes, depressed mood, and irritability are more frequent and/or more severe in women, especially in prodromal and early-manifest stages [6, 8, 34, 35, 37]. Women in the general population have higher rates of depression than men, yet evidence in HD suggests a possible disease-related effect in females, with elevated prevalence and burden of depressive symptoms beyond background epidemiology [62]. To disentangle baseline sex differences from HD-specific vulnerability, larger longitudinal studies with appropriately matched healthy control groups are needed to contextualize these findings. Nevertheless, this pattern has direct therapeutic implications: because psychiatric symptoms often precede motor onset, women with HD may benefit from earlier and more proactive implementation of psychotherapy and/or psychiatric pharmacotherapy [63]. Crucially, the higher baseline risk of depression in women should not be used to normalize or downplay mood symptoms in female HD gene carriers; rather, the elevated prevalence in HD underscores the need for systematic screening and timely diagnosis in this group.

Consistent across genetic studies is the observation that CAG repeat length does not differ between male and female HD patients, when the transmitting parent is not accounted for. This has been shown repeatedly in medium and large cohorts [6, 7, 8, 37] indicating that hormonal, behavioral, and psychosocial influences may underlie these differences or genetic modifiers that have not yet been analyzed.

When considering intergenerational transmission, distinct sex-specific mechanisms become apparent. Paternal transmission is strongly associated with CAG repeat anticipation and earlier disease onset, whereas maternal transmission more often results in CAG repeat stability or contraction, a pattern replicated across multiple independent cohorts [20–23, 24, 25]. These transmission dynamics do not currently translate into sex-tailored pharmacological treatment, but they are highly relevant for genetic counseling and patient education, particularly when discussing recurrence risks, age-at-onset expectations, and the likelihood of juvenile disease in offspring.

### Limitations

Our scoping review has some limitations. We only searched two electronic databases – PubMed and Google Scholar. Since Google Scholar returned over 9,000 results and the first 500 screened by relevance filter covered sufficient relevant studies, this should be adequate for the scope of this review. Nevertheless, we cannot rule out that some studies may have been missed by not including additional databases. Another limitation arises from methodological inconsistencies in the included studies, where confounders, such as medication, comorbidities or age have not been included in the study statistics and therefore could distort results especially in the phenomenology of the disease.

## Conclusions

In this scoping review, we summarized the existing studies regarding sex-related differences in HD. Despite equal genetic risk, sex-related differences in HD are repeatedly reported across several domains. The most consistent evidence concerns intergenerational transmission and clinical phenotype, where women more often exhibit depression and/or irritability and greater functional impact, while men more frequently present with apathy and substance misuse. These differences are also reflected in treatment patterns, with higher antidepressant/anxiolytic use in women and more antipsychotic exposure in men.

At the same time, the literature is heterogeneous in design, outcomes, and adjustment for confounders, and biomarker findings remain preliminary, with signals suggesting sex-specific variation in body composition, uric acid, neuroimaging measures, and hormonal profiles but limited replication.

Future studies should routinely apply sex-stratified analyses, use harmonized longitudinal outcome frameworks, and account for key modifiers such as medication exposure, disease stage, and psychosocial factors.

## Supplementary Information

Supplementary Material 1

## Data Availability

No datasets were generated or analysed during the current study.

## References

[1] McColgan P, Tabrizi SJ. Huntington’s disease: a clinical review. Eur J Neurol. 2018;25:24–34. 28817209 10.1111/ene.13413

[2] Ross CA, Tabrizi SJ. Huntington’s disease: from molecular pathogenesis to clinical treatment. Lancet Neurol. 2011;10:83–98. 21163446 10.1016/S1474-4422(10)70245-3

[3] Walker FO. Huntington’s disease. Lancet. 2007;369:218–28. 17240289 10.1016/S0140-6736(07)60111-1

[4] Kaufhold CJ, Mani KK, Akbari Z, et al. Sex differences in neurological disorders: insights from ischemic stroke, Parkinson’s disease, and multiple sclerosis. Brain Behav Immun. 2025;129:335–47. 40553933 10.1016/j.bbi.2025.06.026

[5] Solla P, Cannas A, Ibba FC, et al. Gender differences in motor and non-motor symptoms among Sardinian patients with Parkinson’s disease. J Neurol Sci. 2012;323:33–9. 22935408 10.1016/j.jns.2012.07.026

[6] Zielonka D, Marinus J, Roos RAC, et al. The influence of gender on phenotype and disease progression in patients with Huntington’s disease. Parkinsonism Relat Disord. 2013;19:192–7. 23102616 10.1016/j.parkreldis.2012.09.012

[7] Zielonka D, Ren M, De Michele G, et al. The contribution of gender differences in motor, behavioral and cognitive features to functional capacity, independence and quality of life in patients with Huntington’s disease. Parkinsonism Relat Disord. 2018;49:42–7. 29326033 10.1016/j.parkreldis.2018.01.006

[8] Hentosh S, Zhu L, Patino J, et al. Sex differences in Huntington’s disease: evaluating the Enroll-HD database. Mov Disord Clin Pract. 2021;8:420–6. 33816672 10.1002/mdc3.13178 PMC8015889

[9] Risby-Jones G, Lee JD, Woodruff TM, et al. Sex differences in Huntington’s disease from a neuroinflammation perspective. Front Neurol. 2024;15:1384480. 38915800 10.3389/fneur.2024.1384480 PMC11194371

[10] Meoni S, Macerollo A, Moro E. Sex differences in movement disorders. Nat Rev Neurol. 2020;16:84–96. 31900464 10.1038/s41582-019-0294-x

[11] Baig SS, Strong M, Rosser E, et al. 22 years of predictive testing for Huntington’s disease: the experience of the UK Huntington’s prediction consortium. Eur J Hum Genet. 2016;24:1396–402. 27165004 10.1038/ejhg.2016.36 PMC5027682

[12] Farrer LA, Cupples LA, Kiely DK, et al. Inverse relationship between age at onset of Huntington disease and paternal age suggests involvement of genetic imprinting. Am J Hum Genet. 1992;50:528–35. 1531729 PMC1684271

[13] Myers RH, Sax DS, Koroshetz WJ, et al. Factors associated with slow progression in Huntington’s disease. Arch Neurol. 1991;48:800–4. 1832854 10.1001/archneur.1991.00530200036015

[14] Roos RA, Vegter-van Der Vlis M, Hermans J, et al. Age at onset in Huntington’s disease: effect of line of inheritance and patient’s sex. J Med Genet. 1991;28:515–9. 1833547 10.1136/jmg.28.8.515 PMC1016978

[15] Medina A, Mahjoub Y, Shaver L, et al. Prevalence and Incidence of Huntington’s Disease: An Updated Systematic Review and Meta-Analysis. Mov Disord. 2022;37:2327–35. 36161673 10.1002/mds.29228 PMC10086981

[16] Bruzelius E, Scarpa J, Zhao Y, et al. Huntington’s disease in the United States: variation by demographic and socioeconomic factors. Mov Disord. 2019;34:858–65. 30868663 10.1002/mds.27653 PMC6579693

[17] Saft C, Andrich J, Brune N, et al. Apolipoprotein E genotypes do not influence the age of onset in Huntington’s disease. J Neurol Neurosurg Psychiatry. 2004;75:1692–6. 15548484 10.1136/jnnp.2003.022756 PMC1738834

[18] Dale M, Maltby J, Shimozaki S, et al. Disease stage, but not sex, predicts depression and psychological distress in Huntington’s disease: A European population study. J Psychosom Res. 2016;80:17–22. 26721543 10.1016/j.jpsychores.2015.11.003

[19] Zielonka D. Gender differences in the CAG repeats and clinical picture correlations in Huntington’s disease. Cesk Slov Neurol Neurochir. 2008;71:688–94.

[20] Aziz NA, Van Belzen MJ, Coops ID, et al. Parent-of-origin differences of mutant HTT CAG repeat instability in Huntington’s disease. Eur J Med Genet. 2011;54:e413–8. 21540131 10.1016/j.ejmg.2011.04.002

[21] Goldberg YP, Kremer B, Andrew SE, et al. Molecular analysis of new mutations for Huntington’s disease: intermediate alleles and sex of origin effects. Nat Genet. 1993;5:174–9. 8252043 10.1038/ng1093-174

[22] Kremer B, Almqvist E, Theilmann J, et al. Sex-dependent mechanisms for expansions and contractions of the CAG repeat on affected Huntington disease chromosomes. Am J Hum Genet. 1995;57:343–50. 7668260 PMC1801544

[23] Zühlke C, Rless O, Schröder K, et al. Expansion of the (CAG)n repeat causing Huntington’s disease in 352 patients of German origin. Hum Mol Genet. 1993;2:1467–9. 8242072 10.1093/hmg/2.9.1467

[24] Trottier Y, Biancalana V, Mandel JL. Instability of CAG repeats in Huntington’s disease: relation to parental transmission and age of onset. J Med Genet. 1994;31:377–82. 8064815 10.1136/jmg.31.5.377 PMC1049869

[25] Nørremølle A, Sørensen SA, Fenger K, et al. Correlation between magnitude of CAG repeat length alterations and length of the paternal repeat in paternally inherited Huntington’s disease. Clin Genet. 1995;47:113–7. 7634532 10.1111/j.1399-0004.1995.tb03941.x

[26] Cannella M, Gellera C, Maglione V, et al. The gender effect in juvenile Huntington disease patients of Italian origin. Am J Med Genet B Neuropsychiatr Genet. 2004;125B:92–8. 14755452 10.1002/ajmg.b.20110

[27] Telenius H, Kremer HPH, Thellmann J, et al. Molecular analysis of juvenile Huntington disease: the major influence on (CAG)n repeat length is the sex of the affected parent. Hum Mol Genet. 1993;2:1535–40. 8268906 10.1093/hmg/2.10.1535

[28] Ranen NG, Stine OC, Abbott MH, et al. Anticipation and Instability of IT-15 (CAG)N Repeats in Parent-Offspring Pairs with Huntington Disease. Am J Hum Genet. 1995;57:593–602. 7668287 PMC1801258

[29] Wheeler VC, Persichetti F, McNeil SM, et al. Factors associated with HD CAG repeat instability in Huntington disease. J Med Genet. 2007;44:695–701. 17660463 10.1136/jmg.2007.050930 PMC2705129

[30] Foroud T, Gray J, Ivashina J, et al. Differences in duration of Huntington’s disease based on age at onset. J Neurol Neurosurg Psychiatry. 1999;66:52–6. 9886451 10.1136/jnnp.66.1.52 PMC1736160

[31] Weydt P, Soyal SM, Landwehrmeyer GB, et al. A single nucleotide polymorphism in the coding region of PGC-1α is a male-specific modifier of Huntington disease age-at-onset in a large European cohort. BMC Neurol. 2014;14:1. 24383721 10.1186/1471-2377-14-1 PMC3880172

[32] Kehoe P, Krawczak M, Harper P, et al. Age of onset in Huntington disease: sex specific influence of apolipoprotein E genotype and normal CAG repeat length. J Med Genet. 1999;36:108–11. 10051007 PMC1734310

[33] Taylor S. Gender differences in attitudes among those at risk for Huntington’s disease. Genet Test. 2005;9:152–7. 15943556 10.1089/gte.2005.9.152

[34] Ekkel MR, Veenhuizen RB, van Loon AM, et al. Nursing home residents with Huntington’s disease: heterogeneity in characteristics and functioning. Brain Cogn. 2023;169:106002. 37269816 10.1016/j.bandc.2023.106002

[35] Epping EA, Mills JA, Beglinger LJ, et al. Characterization of depression in prodromal Huntington disease in the neurobiological predictors of HD (PREDICT-HD) study. J Psychiatr Res. 2013;47:1423–31. 23790259 10.1016/j.jpsychires.2013.05.026 PMC3808084

[36] Levkova M, Tsalta-Mladenov M, Stoyanova M, et al. Two Decades of Huntington’s Disease in Varna, Bulgaria: A Retrospective Single-Centre Study of Clinical Trends and Challenges. Neurol Int. 2025;17:95. 40559333 10.3390/neurolint17060095 PMC12195989

[37] Hemicker G, Schwarzová K, Labrecque S, et al. Bridging the gap: sex-specific differences in Huntington’s disease. Orphanet J Rare Dis. 2026(1). 10.1186/s13023-025-04184-3. 10.1186/s13023-025-04184-3 PMC12882381 41520110

[38] Van Duijn E. Correlates of apathy in Huntington’s disease. J Neuropsychiatry Clin Neurosci. 2010. 10.1176/jnp.2010.22.3.287. 20686135 10.1176/jnp.2010.22.3.287

[39] Marder K, Zhao H, Myers RH, et al. Rate of functional decline in Huntington’s disease. Neurology. 2000;54:452–452. 10668713 10.1212/wnl.54.2.452

[40] Chen S, Zhang H, Yu J et al. May. Sex-Specific Differences in the Progression of Huntington’s Disease Symptoms: A National Study in China. *Neuroepidemiology*. Epub ahead of print 30 2024. 10.1159/000539131 10.1159/000539131 PMC11797925 38815560

[41] Feleus S, Skotnicki LEM, Roos RAC, et al. Medication use and treatment indications in Huntington’s disease; analyses from a large cohort. Mov Disord Clin Pract. 2024;11:1530–41. 39431460 10.1002/mdc3.14230 PMC11647978

[42] Rowe KC, Paulsen JS, Langbehn DR, et al. Patterns of serotonergic antidepressant usage in prodromal Huntington disease. Psychiatry Res. 2012;196:309–14. 22397915 10.1016/j.psychres.2011.09.005 PMC3763706

[43] Zeun P, Scahill RI, Tabrizi SJ, et al. Fluid and imaging biomarkers for Huntington’s disease. Mol Cell Neurosci. 2019;97:67–80. 30807825 10.1016/j.mcn.2019.02.004

[44] Costa De Miranda R, Di Lorenzo N, Andreoli A, et al. Body composition and bone mineral density in Huntington’s disease. Nutrition. 2019;59:145–9. 30468934 10.1016/j.nut.2018.08.005

[45] Goodman AOG, Barker RA. Body composition in premanifest Huntington’s disease reveals lower bone density compared to controls. PLoS Curr. 2011;3:RRN1214. 21379361 10.1371/currents.RRN1214 PMC3047010

[46] Corey-Bloom J, Haque A, Aboufadel S, et al. Uric Acid as a Potential Peripheral Biomarker for Disease Features in Huntington’s Patients. Front Neurosci. 2020;14:73. 32194366 10.3389/fnins.2020.00073 PMC7065265

[47] Lee JK, Ding Y, Conrad AL, et al. Sex-Specific Effects of the Huntington Gene on Normal Neurodevelopment. J Neurosci Res. 2017;95:398–408. 27870408 10.1002/jnr.23980 PMC5729280

[48] Looi JCL, Rajagopalan P, Walterfang M, et al. Differential putaminal morphology in Huntington’s disease, frontotemporal dementia and Alzheimer’s disease. Aust N Z J Psychiatry. 2012;46:1145–58. 22990433 10.1177/0004867412457224 PMC4113021

[49] Yao J, Feng G, Shao G, et al. Interplay between sex and Cytosine-Adenine-Guanine-age product score in Huntington’s disease: clinical and neuroimaging perspectives. Mov Disord. 2025. 10.1002/mds.70064. 40985165 10.1002/mds.70064 PMC12951279

[50] Zhang Y, Long JD, Mills JA, et al. Indexing disease progression at study entry with individuals at-risk for Huntington disease. Am J Med Genet B Neuropsychiatr Genet. 2011;156:751–63. 10.1002/ajmg.b.31232 PMC3174494 21858921

[51] Philpott AL, Cummins TDR, Bailey NW, et al. Cortical inhibitory deficits in Huntington’s disease are not influenced by gender. Psychiatry Research: Neuroimaging. 2016;257:1–4. 27685894 10.1016/j.pscychresns.2016.04.018

[52] Sampedro F, Martinez-Horta S, Pérez-Pérez J, et al. Interaction between sex and neurofilament light chain on brain structure and clinical severity in Huntington’s disease. Ann Clin Transl Neurol. 2021;8:2309–13. 34761569 10.1002/acn3.51460 PMC8670315

[53] Unified Huntington’s. disease rating scale: Reliability and consistency. Mov Disord. 1996;11:136–42. 8684382 10.1002/mds.870110204

[54] Bode FJ, Stephan M, Suhling H, et al. Sex differences in a transgenic rat model of Huntington’s disease: decreased 17β-estradiol levels correlate with reduced numbers of DARPP32 + neurons in males. Hum Mol Genet. 2008;17:2595–609. 18502785 10.1093/hmg/ddn159

[55] Túnez I, Collado JA, Medina FJ. 17 β-estradiol may affect vulnerability of striatum in a 3-nitropropionic acid-induced experimental model of Huntington’s disease in ovariectomized rats. Neurochem Int. 2006;48:367–73. 16420966 10.1016/j.neuint.2005.11.011

[56] Markianos M, Panas M, Kalfakis N, et al. Plasma testosterone in male patients with Huntington’s disease: relations to severity of illness and dementia. Ann Neurol. 2005;57:520–5. 15786456 10.1002/ana.20428

[57] Markianos M, Panas M, Kalfakis N et al. Plasma testosterone, dehydroepiandrosterone sulfate, and cortisol in female patients with Huntington’s disease. Neuro Endocrinol Lett. 2007 Apr;28(2):199–203. 17435662

[58] Saleh N, Moutereau S. High insulinlike growth factor I is associated with cognitive decline in Huntington disease. Neurology. 2010. 10.1212/WNL.0b013e3181e62076. 20603485 10.1212/WNL.0b013e3181e62076

[59] Cao Z, Min J, Tan Q, et al. Circulating insulin-like growth factor-1 and brain health: evidence from 369,711 participants in the UK Biobank. Alzheimers Res Ther. 2023;15:140. 37608387 10.1186/s13195-023-01288-5 PMC10463341

[60] Bernstein SR, Kelleher C, Khalil RA. Gender-based research underscores sex differences in biological processes, clinical disorders and pharmacological interventions. Biochem Pharmacol. 2023;215:115737. 37549793 10.1016/j.bcp.2023.115737 PMC10587961

[61] Mauvais-Jarvis F, Bairey Merz N, Barnes PJ, et al. Sex and gender: modifiers of health, disease, and medicine. Lancet. 2020;396:565–82. 32828189 10.1016/S0140-6736(20)31561-0 PMC7440877

[62] Kuehner C. Why is depression more common among women than among men? Lancet Psychiatry. 2017;4:146–58. 27856392 10.1016/S2215-0366(16)30263-2

[63] Bates GP, Dorsey R, Gusella JF, et al. Huntington disease. Nat Rev Dis Primers. 2015;1:15005. 27188817 10.1038/nrdp.2015.5

