# Supplementary material for: Sex-related differences in Huntington‘s disease: a scoping review
Source: Biol Sex Differ. 2026 Apr 3;17:106. doi: 10.1186/s13293-026-00895-9 (PMC13173938; doi:10.1186/s13293-026-00895-9)
Supplement: Supplementary file 1 — Supplementary Material 1 [file 13293_2026_895_MOESM1_ESM.docx]

Table 3: Quality assessment of single case reports using JBI critical appraisal tools for cohort studies ^1^

| **Reference** | **First Author** | **Q1** | **Q2** | **Q3** | **Q4** | **Q5** | **Q6** | **Q7** | **Q8** | **Q9** | **Q10** | **Q11** | **Score** | **Quality (%)** | **Status** |
| --- | --- | --- | --- | --- | --- | --- | --- | --- | --- | --- | --- | --- | --- | --- | --- |
| ^2^ | Bruzelius | N/A | 1 | 1 | 1 | 1 | N/A | 1 | 1 | N/A | N/A | 1 | 7 | 100% | high |
| ^3^ | Foroud | 1 | 1 | 1 | 1 | N/A | 1 | 1 | 1 | N/A | N/A | 1 | 8 | 100% | high |
| ^4^ | Hentosh | 1 | 1 | 1 | 1 | 1 | 1 | 1 | 1 | 1 | 0 | 1 | 10 | 91% | high |
| ^5^ | Kremer | 1 | 1 | 1 | 0 | 0 | 1 | 1 | N/A | N/A | N/A | 1 | 6 | 75% | high |
| ^6^ | Lee | 1 | 1 | 1 | 1 | 1 | 1 | 1 | 1 | 1 | 1 | 1 | 11 | 100% | high |
| ^7^ | Marder | 0 | 0 | 1 | 1 | 1 | 0 | 1 | 1 | 0 | 0 | 1 | 6 | 55% | medium |
| ^8^ | Myers | 1 | 1 | 1 | 1 | 1 | 1 | 1 | 1 | N/A | N/A | 1 | 9 | 100% | high |
| ^9^ | Nørremølle | 1 | 1 | 1 | 0 | 0 | 1 | 1 | N/A | N/A | N/A | 1 | 6 | 67% | high |
| ^10^ | Ranen | 1 | 1 | 1 | 1 | 1 | 1 | 1 | N/A | N/A | N/A | 1 | 8 | 89% | high |
| ^11^ | Roos | 1 | 1 | 1 | 0 | 0 | 1 | 1 | 1 | N/A | N/A | 1 | 7 | 78% | high |
| ^12^ | Saleh | 1 | 1 | 1 | 1 | 1 | 1 | 1 | 1 | 0 | 0 | 1 | 9 | 82% | high |
| ^13^ | Yao | 1 | 1 | 1 | 1 | 1 | 1 | 1 | 1 | 1 | 1 | 1 | 11 | 100% | high |
| ^14^ | Zielonka (2013) | 1 | 1 | 1 | 1 | 1 | 1 | 1 | 1 | 0 | 0 | 1 | 9 | 82% | high |

1 = quality criterion fulfilled

0 = quality criterion not fulfilled

N/A = not applicable

Table 4: Quality assessment of single case reports using JBI critical appraisal tools for cross-sectional studies

| **Reference** | **First Author** | **Q1** | **Q2** | **Q3** | **Q4** | **Q5** | **Q6** | **Q7** | **Q8** | **Score** | **Quality (%)** | **Status** |
| --- | --- | --- | --- | --- | --- | --- | --- | --- | --- | --- | --- | --- |
| ^15^ | Aziz | 0 | 0 | 1 | 1 | 1 | 1 | 1 | 1 | 8 | 100% | high |
| ^16^ | Cannella | 1 | 1 | 1 | 1 | 1 | 1 | 1 | 1 | 8 | 100% | high |
| ^17^ | Chen | 1 | 1 | 0 | 1 | 0 | 0 | 1 | 1 | 5 | 63% | medium |
| ^18^ | Corey-Bloom | 1 | 1 | 1 | 1 | 1 | 1 | 1 | 1 | 8 | 100% | high |
| ^19^ | Costa De Miranda | 1 | 1 | 1 | 1 | 1 | 1 | 1 | 1 | 8 | 100% | high |
| ^20^ | Dale | 1 | 1 | 1 | 1 | 1 | 1 | 1 | 1 | 8 | 86% | high |
| ^21^ | Ekkel | 1 | 1 | 1 | 1 | 0 | 0 | 1 | 1 | 6 | 75% | high |
| ^22^ | Epping | 1 | 1 | 1 | 1 | 1 | 1 | 1 | 1 | 8 | 100& | high |
| ^23^ | Farrer | 1 | 1 | 1 | 1 | 1 | 1 | 1 | 1 | 8 | 100% | high |
| ^24^ | Feleus | 1 | 1 | 1 | 1 | 1 | 1 | 1 | 1 | 8 | 100% | high |
| ^25^ | Goodman & Barker | 1 | 1 | 1 | 1 | 1 | 1 | 1 | 1 | 8 | 100% | high |
| ^26^ | Hemicker | 1 | 1 | 1 | 1 | 1 | 1 | 1 | 1 | 8 | 100% | high |
| ^27^ | Kehoe | 1 | 1 | 1 | 1 | 1 | 1 | 1 | 1 | 8 | 100% | high |
| ^28^ | Levkova | 1 | 1 | 1 | 1 | 0 | 0 | 1 | 1 | 6 | 75% | high |
| ^29^ | Looi | 1 | 1 | 1 | 1 | 1 | 1 | 1 | 1 | 8 | 100% | high |
| ^30^ | Rowe | 1 | 1 | 1 | 1 | 1 | 1 | 1 | 1 | 8 | 100% | high |
| ^31^ | Saft | 1 | 1 | 1 | 1 | 1 | 1 | 1 | 1 | 8 | 100% | high |
| ^32^ | Sampedro | 1 | 1 | 1 | 1 | 1 | 1 | 1 | 1 | 8 | 100% | high |
| ^33^ | Taylor | 1 | 1 | 1 | 1 | 0 | 0 | 1 | 1 | 6 | 75% | high |
| ^34^ | Telenius | 1 | 1 | 1 | 1 | 0 | 0 | 1 | 1 | 6 | 75% | high |
| ^35^ | Trottier | 1 | 1 | 1 | 1 | 0 | 0 | 1 | 1 | 6 | 75% | high |
| ^36^ | Van Duijn | 1 | 1 | 1 | 1 | 1 | 1 | 1 | 1 | 8 | 100% | high |
| ^37^ | Weydt | 1 | 1 | 1 | 1 | 1 | 1 | 1 | 1 | 8 | 100% | high |
| ^38^ | Wheeler | 1 | 1 | 1 | 1 | 1 | 1 | 1 | 1 | 8 | 100% | high |
| ^39^ | Zielonka (2008) | 1 | 1 | 1 | 1 | 0 | 0 | 1 | 1 | 6 | 75% | high |
| ^40^ | Zielonka (2018) | 1 | 1 | 1 | 1 | 1 | 1 | 1 | 1 | 8 | 100% | high |

1 = quality criterion fulfilled

0 = quality criterion not fulfilled

N/A = not applicable

Table 5: Quality assessment of single case reports using JBI critical appraisal tools for case series

| **Reference** | **First Author** | **Q1** | **Q2** | **Q3** | **Q4** | **Q5** | **Q6** | **Q7** | **Q8** | **Q9** | **Q10** | **Score** | **Quality (%)** | **Status** |
| --- | --- | --- | --- | --- | --- | --- | --- | --- | --- | --- | --- | --- | --- | --- |
| ^41^ | Goldberg | 1 | 1 | 1 | 0 | 1 | 1 | 1 | N/A | 1 | 1 | 8 | 80% | high |

1 = quality criterion fulfilled

0 = quality criterion not fulfilled

N/A = not applicable

Table 6: Quality assessment of case control studies using JBI critical appraisal tools for case control studies

| **Reference** | **First Author** | **Q1** | **Q2** | **Q3** | **Q4** | **Q5** | **Q6** | **Q7** | **Q8** | **Q9** | **Q10** | **Score** | **Quality (%)** | **Status** |
| --- | --- | --- | --- | --- | --- | --- | --- | --- | --- | --- | --- | --- | --- | --- |
| ^42^ | Markianos (2005) | 1 | 1 | 1 | 1 | 1 | 1 | 1 | 1 | 1 | 1 | 10 | 100% | high |
| ^43^ | Markianos (2007) | 1 | 1 | 1 | 1 | 1 | 1 | 1 | 1 | 1 | 1 | 10 | 100% | high |
| ^44^ | Zühlke | 0 | 0 | 1 | 1 | 1 | 0 | 0 | 1 | 0 | 1 | 5 | 50% | medium |
| ^45^ | Philpott | 1 | 1 | 1 | 1 | 1 | 1 | 1 | 1 | 1 | 1 | 10 | 100% | high |

1 = quality criterion fulfilled

0 = quality criterion not fulfilled

N/A = not applicable

Figure 1: Flowchart of the study selection process

References

1. Porritt K, Gomersall J, Lockwood C. JBI’s Systematic Reviews: Study selection and critical appraisal. *Am J Nurs* 2014; 114: 47–52.

2. Bruzelius E, Scarpa J, Zhao Y, et al. Huntington’s Disease in the United States: Variation by demographic and socioeconomic factors. *Mov Disord Off J Mov Disord Soc* 2019; 34: 858–865.

3. Foroud T, Gray J, Ivashina J, et al. Differences in duration of Huntington’s disease based on age at onset. *J Neurol Neurosurg Psychiatry* 1999; 66: 52–56.

4. Hentosh S, Zhu L, Patino J, et al. Sex Differences in Huntington’s Disease: Evaluating the Enroll‐HD Database. *Mov Disord Clin Pract* 2021; 8: 420–426.

5. Kremer B, Almqvist E, Theilmann J, et al. Sex-dependent mechanisms for expansions and contractions of the CAG repeat on affected Huntington disease chromosomes. *Am J Hum Genet* 1995; 57: 343–350.

6. Lee JK, Ding Y, Conrad AL, et al. Sex-Specific Effects of the Huntington Gene on Normal Neurodevelopment. *J Neurosci Res* 2017; 95: 398–408.

7. Marder K, Zhao H, Myers RH, et al. Rate of functional decline in Huntington’s disease. *Neurology* 2000; 54: 452–452.

8. Myers RH, Sax DS, Koroshetz WJ, et al. Factors Associated With Slow Progression in Huntington’s Disease. *Arch Neurol* 1991; 48: 800–804.

9. Nørremølle A, Sørensen SA, Fenger K, et al. Correlation between magnitude of CAG repeat length alterations and length of the paternal repeat in paternally inherited Huntington’s disease. *Clin Genet* 1995; 47: 113–117.

10. Ranen NG, Stine OC, Abbott MH, et al. Anticipation and Instability of IT-15 (CAG)N Repeats in Parent-Offspring Pairs with Huntington Disease. *Am J Hum Genet* 1995; 57: 593–602.

11. Roos RA, Vegter-van Der Vlis M, Hermans J, et al. Age at onset in Huntington’s disease: effect of line of inheritance and patient’s sex. *J Med Genet* 1991; 28: 515–519.

12. Saleh N, Moutereau S. High insulinlike growth factor I is associated with cognitive decline in Huntington disease. *Neurology*.

13. Yao J, Feng G, Shao G, et al. Interplay Between Sex and Cytosine-Adenine-Guanine-Age Product Score in Huntington’s Disease: Clinical and Neuroimaging Perspectives. *Mov Disord*; n/a. Epub ahead of print 2025. DOI: 10.1002/mds.70064.

14. Zielonka D, Marinus J, Roos RAC, et al. The influence of gender on phenotype and disease progression in patients with Huntington’s disease. *Parkinsonism Relat Disord* 2013; 19: 192–197.

15. Aziz NA, Van Belzen MJ, Coops ID, et al. Parent-of-origin differences of mutant HTT CAG repeat instability in Huntington’s disease. *Eur J Med Genet* 2011; 54: e413–e418.

16. Cannella M, Gellera C, Maglione V, et al. The gender effect in juvenile Huntington disease patients of Italian origin. *Am J Med Genet B Neuropsychiatr Genet* 2004; 125B: 92–98.

17. Chen S, Zhang H, Yu J, et al. Sex-Specific Differences in the Progression of Huntington’s Disease Symptoms: A National Study in China. *Neuroepidemiology*. Epub ahead of print 30 May 2024. DOI: 10.1159/000539131.

18. Corey-Bloom J, Haque A, Aboufadel S, et al. Uric Acid as a Potential Peripheral Biomarker for Disease Features in Huntington’s Patients. *Front Neurosci* 2020; 14: 73.

19. Costa De Miranda R, Di Lorenzo N, Andreoli A, et al. Body composition and bone mineral density in Huntington’s disease. *Nutrition* 2019; 59: 145–149.

20. Dale M, Maltby J, Shimozaki S, et al. Disease stage, but not sex, predicts depression and psychological distress in Huntington’s disease: A European population study. *J Psychosom Res* 2016; 80: 17–22.

21. Ekkel MR, Veenhuizen RB, van Loon AM, et al. Nursing home residents with Huntington’s disease: Heterogeneity in characteristics and functioning. *Brain Cogn* 2023; 169: 106002.

22. Epping EA, Mills JA, Beglinger LJ, et al. Characterization of depression in prodromal Huntington disease in the neurobiological predictors of HD (PREDICT-HD) study. *J Psychiatr Res* 2013; 47: 1423–1431.

23. Farrer LA, Cupples LA, Kiely DK, et al. Inverse relationship between age at onset of Huntington disease and paternal age suggests involvement of genetic imprinting. *Am J Hum Genet* 1992; 50: 528–535.

24. Feleus S, Skotnicki LEM, Roos RAC, et al. Medication Use and Treatment Indications in Huntington’s Disease; Analyses from a Large Cohort. *Mov Disord Clin Pract* 2024; 11: 1530–1541.

25. Goodman AOG, Barker RA. Body composition in premanifest Huntington’s disease reveals lower bone density compared to controls. *PLoS Curr* 2011; 3: RRN1214.

26. Hemicker G, Schwarzová K, Labrecque S, et al. Bridging the gap: sex-specific differences in Huntington’s disease. *Orphanet J Rare Dis*. Epub ahead of print 10 January 2026. DOI: 10.1186/s13023-025-04184-3.

27. Kehoe P, Krawczak M, Harper P, et al. Age of onset in Huntington disease: sex specific influence of apolipoprotein E genotype and normal CAG repeat length. *J Med Genet* 1999; 36: 108–111.

28. Levkova M, Tsalta-Mladenov M, Stoyanova M, et al. Two Decades of Huntington’s Disease in Varna, Bulgaria: A Retrospective Single-Centre Study of Clinical Trends and Challenges. *Neurol Int* 2025; 17: 95.

29. Looi JCL, Rajagopalan P, Walterfang M, et al. Differential putaminal morphology in Huntington’s disease, Frontotemporal dementia and Alzheimer’s disease. *Aust N Z J Psychiatry* 2012; 46: 1145–1158.

30. Rowe KC, Paulsen JS, Langbehn DR, et al. Patterns of serotonergic antidepressant usage in prodromal Huntington disease. *Psychiatry Res* 2012; 196: 309–314.

31. Saft C, Andrich J, Brune N, et al. Apolipoprotein E genotypes do not influence the age of onset in Huntington’s disease. *J Neurol Neurosurg Psychiatry* 2004; 75: 1692–1696.

32. Sampedro F, Martinez-Horta S, Pérez-Pérez J, et al. Interaction between sex and neurofilament light chain on brain structure and clinical severity in Huntington’s disease. *Ann Clin Transl Neurol* 2021; 8: 2309–2313.

33. Taylor S. Gender Differences in Attitudes Among Those at Risk for Huntington’s Disease. *Genet Test* 2005; 9: 152–157.

34. Telenius H, Kremer HPH, Thellmann J, et al. Molecular analysis of juvenile Huntington disease: the major influence on (CAG)n repeat length is the sex of the affected parent. *Hum Mol Genet* 1993; 2: 1535–1540.

35. Trottier Y, Biancalana V, Mandel JL. Instability of CAG repeats in Huntington’s disease: relation to parental transmission and age of onset. *J Med Genet* 1994; 31: 377–382.

36. Van Duijn E. Correlates of Apathy in Huntington’s Disease. Epub ahead of print 2010. DOI: 10.1176/jnp.2010.22.3.287.

37. Weydt P, Soyal SM, Landwehrmeyer GB, et al. A single nucleotide polymorphism in the coding region of PGC-1α is a male-specific modifier of Huntington disease age-at-onset in a large European cohort. *BMC Neurol* 2014; 14: 1.

38. Wheeler VC, Persichetti F, McNeil SM, et al. Factors associated with HD CAG repeat instability in Huntington disease. *J Med Genet* 2007; 44: 695–701.

39. Zielonka D. Gender Differences in the CAG Repeats and Clinical Picture Correlations in Huntington’s Disease. *Cesk Slov Neurol* 2008; 71: 688–694.

40. Zielonka D, Ren M, De Michele G, et al. The contribution of gender differences in motor, behavioral and cognitive features to functional capacity, independence and quality of life in patients with Huntington’s disease. *Parkinsonism Relat Disord* 2018; 49: 42–47.

41. Goldberg YP, Kremer B, Andrew SE, et al. Molecular analysis of new mutations for Huntington’s disease: intermediate alleles and sex of origin effects. *Nat Genet* 1993; 5: 174–179.

42. Markianos M, Panas M, Kalfakis N, et al. Plasma testosterone in male patients with Huntington’s disease: Relations to severity of illness and dementia. *Ann Neurol* 2005; 57: 520–525.

43. Markianos M, Panas M, Kalfakis N, et al. Plasma testosterone, dehydroepiandrosterone sulfate, and cortisol in female patients with Huntington’s disease. *Neuro Endocrinol Lett*.

44. Zühlke C, Rless O, Schröder K, et al. Expansion of the (CAG)n repeat causing Huntington’s disease in 352 patients of German origin. *Hum Mol Genet* 1993; 2: 1467–1469.

45. Philpott AL, Cummins TDR, Bailey NW, et al. Cortical inhibitory deficits in Huntington’s disease are not influenced by gender. *Psychiatry Res Neuroimaging* 2016; 257: 1–4.
